# Social isolation: an integrated molecular web that disrupts cellular homeostasis

**DOI:** 10.3389/fnins.2025.1693696

**Published:** 2025-12-08

**Authors:** Mohammed Qaisiya, Edoardo Moretto, Elisabetta Battocchio, Aurora Pistella, Maria Giuseppa Caso, Marcella Bellani, Fabrizia Claudia Guarnieri

**Affiliations:** 1 Department of Medical Laboratory Science, Hebron University, Hebron, Palestine; 2 CNR Institute of Neuroscience, Vedano al Lambro, Italy; 3 Section of Psychiatry, Department of Neurosciences, Biomedicine and Movement Sciences, University of Verona, Verona, Italy; 4 Unit of Psychiatry, Azienda Ospedaliera Universitaria Integrata (AOUI) Verona, Verona, Italy

**Keywords:** social deprivation, loneliness, metabolism, oxidative stress, inflammation

## Abstract

Social isolation and perceived loneliness are increasingly recognized as serious public health concerns, with extensive evidence linking them to adverse mental and physical health outcomes. Defined, respectively, as the objective lack of social interactions and the subjective feeling of insufficient connection, both conditions are present across various age groups and are associated with elevated risks of cognitive decline and psychiatric disorders. Epidemiological studies have also identified a strong association between chronic social isolation and the development of metabolic syndrome (MetS) and cardiovascular diseases (CVD), potentially mediated by dysregulated stress responses, immune function, and endocrine signaling. Animal models of social deprivation have proven instrumental in elucidating the biological underpinnings of these effects, revealing disruptions in neurotransmitter systems and in the hypothalamic–pituitary–adrenal (HPA) axis, with important downstream metabolic alterations. This review explores the molecular and cellular mechanisms linking social isolation to MetS and CVD, with a focus on oxidative stress, inflammation, mitochondrial dysfunction, and impaired autophagy. A deeper understanding of these pathways is essential to guide the development of targeted interventions and to reduce the long-term health burden associated with social disconnection.

## Introduction

Social isolation and perceived loneliness have recently emerged as public health concerns in modern societies, with a growing recognition of their impact on both mental and physical health. As a highly social species, humans rely on meaningful interpersonal relationships for emotional support, stress regulation, and overall wellbeing. While loneliness is defined as a subjective feeling of insufficient social contact as compared to personal expectations, social isolation refers to an objective state of lack of adequate social interactions, emotional connections, or engagement within a community. These two conditions are distinct yet closely interconnected. The estimated prevalence of social isolation in adolescents and young adults ranges from 5.4% in a German study (18–39 years of age) to 10.14% in Saudi Arabia (18–21 years) and 17% in Switzerland (15–24 years) (Hämmig 2019; Röhr et al., 2022; Alsadoun et al., 2023). It increases to 20% in middle aged adults (25–44 years), 23% in advanced adults (45–64 years) and 21.7%–35% in the elderly (>65 years) (Hämmig 2019; Röhr et al., 2022). Loneliness has an estimated prevalence of 9.2%–14.4% in adolescents (12–17 years of age), with regional variability in different countries worldwide. In Europe, the reported prevalence of loneliness is 2.9%–7.5% in young adults (18–29 years), 2.7%–9.6% in middle aged adults (30–59 years), and 5.2%–21.3% in older adults (>60 years) (Surkalim et al., 2022), reaching up to 27.6% in older adults worldwide (Salari et al., 2025). Thus, despite significant geographical variation in the availability and estimates of prevalence data, social isolation and loneliness impact a considerable fraction of the global population.

A complex interplay of cultural, environmental, and psychosocial factors underlies vulnerability to social isolation and loneliness. These include early-life adversities, exposure to chronic stress, insufficient parental care, and hostile or neglectful social environments during critical periods of neurodevelopment (Vitale and Smith, 2022). Polygenic risk factors contributing to social isolation behaviors have been recently identified (Socrates et al., 2024; Rødevand et al., 2021). These aspects collectively shape social behavior and impact an individual’s capacity to form and maintain relationships.

It is now well-established that prolonged social isolation or loneliness have significant physiological and biological consequences. Objective or perceived social isolation predisposes to the development of cognitive deficits and psychiatric conditions (Cacioppo and Hawkley, 2009; Ren et al., 2023), and is associated with a 25%–30% increased risk of mortality (Laugesen et al., 2018; Holt-Lunstad et al., 2015). Alterations in stress reactivity, immune function and neuroendocrine regulation have all been observed, contributing to an increased incidence of diabetes, metabolic syndrome (MetS) and cardiovascular diseases (CVD) in isolated or lonely subjects (Sharma et al., 2021; Brinkhues et al., 2017; Ahmed et al., 2023).

Although social isolation in humans is a complex and subjective experience, animal models provide a valuable and controlled framework to dissect its underlying biological mechanisms. Social deprivation induced in rodents recapitulates several behavioral phenotypes observed in the human condition, including anxiety, depressive and stress behaviors, with sex-specific susceptibilities (Zorzo et al., 2019; Walker et al., 2019). In this review, we examine the molecular and cellular mechanisms through which social isolation exerts its pathophysiological effects in both humans and animal models. We focus on how social disconnection disrupts homeostatic systems, particularly those governing stress responses, inflammation, and metabolic regulation, and how these alterations may contribute to the pathophysiology of MetS and CVD in isolated or lonely individuals. Understanding these biological pathways is critical for developing effective interventions and mitigating the severe long-term health consequences of social isolation.

### Clinical association between social isolation and MetS or CVD

Social isolation and chronic loneliness are known to induce a persistent hypervigilant state, characterized by heightened anxiety, increased stress reactivity, and reduced sleep quality, which collectively promote further social withdrawal and maladaptive behaviors such as sedentariness or substance abuse. Psychosocial stressors are known to activate neuroendocrine systems involved in the stress response, including the autonomic nervous system and the hypothalamic–pituitary–adrenal (HPA) axis, which triggers the release of corticotropin-releasing hormone (CRH) from the hypothalamus, adrenocorticotropic hormone (ACTH) from the anterior pituitary gland, and ultimately glucocorticoids, such as cortisol, from the adrenal glands. A negative feedback loop of cortisol on the HPA axis physiologically limits HPA activation and stress hormones release. Several reports have associated social isolation and loneliness with elevated levels of cortisol and a flattening of its diurnal rhythm, suggesting HPA perturbations (Doane and Adam, 2010; Jopling et al., 2021; Grant et al., 2009).

Glucocorticoids, released in a pulsatile way across the day, are critically implicated in the physiological regulation of glucose and lipid homeostasis, inflammation, immune response, and cardiovascular function. Indeed, at the cellular level, glucocorticoids act on nuclear receptors that modulate the transcription of hundreds of genes involved in metabolism and anti-inflammatory response. In the vascular system, glucocorticoids reduce the production of endothelial nitric oxide, thus promoting vasoconstriction. Chronic excess secretion of these hormones can lead to hyperglycemia, insulin resistance, redistribution of body fat mass and hypertension, all of which predispose to the development of MetS and CVD (Kivimäki et al., 2023). Numerous studies have shown that both social isolation and loneliness significantly increase the risk of developing MetS (Henriksen et al., 2019; Delolmo-Romero et al., 2024). Several cohort studies (UK Biobank, CHARLS, and HUNT) demonstrated an association of both social isolation and loneliness with elevated risk of type-2 diabetes mellitus (Song et al., 2023; Henriksen et al., 2023; Asif et al., 2025; Ezzatvar et al., 2025) and non-alcoholic fatty liver disease (Miao et al., 2025). The association of both social isolation and loneliness with increased risk of CVD, including coronary heart disease and stroke, is well documented in adults (Albasheer et al., 2024; Cené et al., 2022; Valtorta et al., 2016; Hakulinen et al., 2018; Bu et al., 2020; Valtorta et al., 2018; Leigh-Hunt et al., 2017; Freak-Poli et al., 2021). Recent reports suggested that early indicators of poor cardiovascular health can be detected in lonely young adults in their twenties (Vasan et al., 2024; Roddick and Chen, 2021).

Perturbations in systemic inflammation have also been described. Several reports associated social isolation and loneliness with increased plasmatic levels of inflammatory markers such as interleukin-6 (IL-6), C-reactive protein (CRP), soluble urokinase plasminogen activator (suPAR), fibrinogen and ferritin (Zilioli and Jiang, 2021; Van Bogart et al., 2021; Matthews et al., 2024; Nersesian et al., 2018; Vingeliene et al., 2019; Loucks et al., 2006; Shankar et al., 2011). In particular, a recent meta-analysis reported that loneliness shows a stronger association with IL-6 levels, whereas social isolation is more closely linked to CRP and fibrinogen, suggesting distinct alterations underlying the two conditions (Smith et al., 2020). Interestingly, few longitudinal studies indicated that social isolation during childhood predicts increased inflammation later in life (Matthews et al., 2024; Lacey et al., 2014). The inflammatory propensity of loneliness is even enhanced in acute stress situations, suggesting atypical stress reactivity in lonely individuals (Hackett et al., 2012; Brown et al., 2018). Gene expression studies on circulating leukocytes further evidenced increased inflammation and immune activation in loneliness (Brown et al., 2020). These effects could be attributed to an increased activity of the pro-inflammatory transcription factors NF-κB (nuclear factor kappa B) and JAK/STAT (Janus kinase/signal transducer and activator of transcription), with a reciprocal reduction in the transcription of anti-inflammatory glucocorticoid receptor target genes in lonely older adults, independently of the objective size of their social network (Cole et al., 2007). This is consistent with reports of glucocorticoid resistance in the context of loneliness, in which glucocorticoid receptors in leukocytes become less effective at transducing cortisol signaling into a transcriptional response. Interestingly, these changes appeared to depend primarily on the subjective perception of isolation rather than on the objective social connectivity (Cole, 2008).

A recent proteome-wide association study performed on a huge cohort of subjects provided new important molecular insight on the possible molecular mechanisms underlying loneliness and social isolation, and on their link to metabolic and cardiovascular complications (Shen et al., 2025). Plasma proteomics and protein network analyses revealed that protein modules involved in immune functions and metabolic processes were the most significantly associated with both social isolation and loneliness. In particular, the inflammatory marker GDF15 (growth differentiation factor 15) and the PCSK9 enzyme (proprotein convertase subtilisin/kexin type 9) involved in cholesterol metabolism were identified as the most strongly associated with social isolation and loneliness, respectively. The immune molecule CXCL14 (C-X-C motif chemokine ligand-14) emerged as a protective factor against social isolation. A causal relationship specifically with loneliness was suggested for five proteins (GDNF receptor alpha 1, GFRA1; adrenomedullin, ADM; fatty acid binding protein 4, FABP4; TNF receptor superfamily member 10A, TNFRSF10A; asialoglycoprotein receptor 1, ASGR1), which exhibited strong correlation with other blood biomarkers such as CRP, as well as with brain volumes of areas involved in emotional and social processing, and had a strong prospective association with CVD, diabetes, stroke and mortality (Shen et al., 2025). Collectively, loneliness and social isolation appear to converge on shared pathological outcomes but also show distinct molecular signatures that deserve further exploration.

Experimental studies performed in animal models (e.g., rats, mice, prairie voles), isolated for hours or up to several weeks, paralleled correlative studies in humans and substantiated a causal role of social isolation in influencing metabolic dysfunctions. Although forced social isolation can yield variable outcomes in different animal species, numerous studies supported the observation that chronic deprivation of interactions results in increased HPA activity, elevated secretion of cortisol (or corticosterone in rodents) and enhanced stress reactivity (Cacioppo et al., 2015). Mice at postnatal day 28 (P28) housed in a thermoneutral environment and isolated for more than 4 weeks showed an increase in body weight and higher circulating leptin and insulin as compared to group-housed litters, even in the absence of detectable corticosterone alterations (Queen et al., 2023). Isolated mice were also more susceptible to obesity-associated metabolic disease when fed with a high-fat diet, showing increased food intake and fat mass, higher blood glucose upon glucose tolerance test, signs of insulin resistance, and increased circulating inflammatory markers (Queen et al., 2023). Accelerated body weight gain and adiposity were also observed in mice genetically predisposed to obesity upon social isolation (Nonogaki et al., 2007). Nevertheless, it has to be noted that others have reported either unaffected or reduced body weight gain in mice isolated for 4 to 6 weeks (Smolensky et al., 2024; Farbstein et al., 2021; Bibancos et al., 2007). Even though contrasting results have been obtained depending on the diet, housing conditions, animal strain and isolation protocol, experimental models have been instrumental in advancing our understanding of the cellular and molecular mechanisms triggered in various brain regions and peripheral tissues by this form of psychosocial stress. The main findings are summarized in the following sections.

### Oxidative stress and inflammation are important features of social isolation

Oxidative stress is the result of an imbalance in pro-oxidant production and cellular antioxidant capacity that leads to excess generation of reactive oxygen species (ROS) or reactive nitrogen species (RNS). The brain is particularly susceptible to oxidative stress, due to its high metabolic rate, lipid content and oxygen consumption (Cobley et al., 2018). Several studies suggested that oxidative stress is a hallmark phenomenon observed in social isolation models due to chronic psychological stress, even though variable results have been obtained depending on sex, starting age and duration of the isolation (Grigoryan et al., 2022; Bijani et al., 2024). Increased expression of the ROS-generating enzymes NADPH oxidases (NOX), elevated levels of ROS, reduced glutathione, and lipid peroxidation have been described in the brain (e.g., hippocampus, prefrontal cortex, and hypothalamus) and in peripheral tissues such as the liver in isolated mice and rats (Bove et al., 2022; Ai et al., 2024; Schiavone et al., 2009; Zlatković et al., 2014). The activity of the antioxidant enzymes catalase, glutathione peroxidase and superoxide dismutase was found to be reduced in various brain areas of rats isolated at weaning for 8 weeks (Shao et al., 2015; Djordjevic J. et al., 2010). Interestingly, experiments on chronically isolated rats demonstrated that treatment with the antioxidant and NOX2 inhibitor apocyanin during the isolation period attenuated HPA axis activation and prevented behavioral alterations (Colaianna et al., 2013; Schiavone et al., 2009). In a recent study where the carpenter ant *Camponotus fellah* was used as an animal model to investigate social isolation, a significant accumulation of ROS was detected in liver-like cells, which was associated with abnormal behaviors and reduced lifespan in isolated ants. The administration of antioxidant compounds significantly extended the lifespan of isolated ants (Koto et al., 2023), demonstrating a causal role of oxidative stress in mediating the detrimental health effects of social isolation.

The transcription factor Nrf2 (nuclear factor erythroid-2 related factor 2) is a master sensor of the cellular redox state. In the absence of stimuli, Nrf2 is retained in the cytoplasm by its inhibitory partner Keap1. Activation of Nrf2 can occur either through direct modification of reactive cysteine residues on Keap1 by ROS or RNS, or indirectly via phosphorylation of Nrf2 by oxidative stress-responsive kinases such as ERK. Once activated, Nrf2 translocates to the nucleus, where it binds to antioxidant response elements (ARE) in the promoters of target genes to restore redox homeostasis. Nrf2-regulated targets include genes coding for proteins involved in glutathione homeostasis (xCT, *γ*-GCLc, GPx, GST, and TRX) and antioxidant response (HO-1, NQO-1, FTH, CAT, SOD, and PRDx), but also autophagy (ATG5, ATG7, and p62/SQSTM1), carbohydrate metabolism (G6PD and ME), and lipid metabolism (ACOT and lipases) (Malhotra et al., 2010; Hayes and Dinkova-Kostova, 2014). As such, the coordinated induction of Nrf2 target genes at the interface between redox and metabolism is a potent adaptive cytoprotective response activated upon various stress conditions (Baird and Dinkova-Kostova, 2011).

Nrf2 activation is a critical protective response against acute and chronic stress (Bouvier et al., 2017; McCallum et al., 2024). Glucocorticoids decrease Nrf2 activity (Alam et al., 2017), suggesting that persistent HPA activation during stress may negatively impact this pathway. Accordingly, an impairment of the Nrf2 pathway has been reported in social isolation. Djordjevic et al. (2015) suggested maladaptive Keap1/Nrf2 signaling in the brain of adult male rats subjected to social isolation for 3 weeks, which showed reduced nuclear Nrf2 levels in the hippocampus, but not in the prefrontal cortex (PFC), as compared to controls with a concomitant increase in nuclear NF-κB (Djordjevic et al., 2015). In a recent study, young male rats were subjected to chronic unpredictable mild stress for 4 weeks followed by 4 weeks of either re-socialization or social isolation. Socially isolated and chronically stressed rats exhibited a significant suppression of the ERK/Keap1/Nrf2 pathway, with reduced phosphorylation of ERK, increased Keap1 expression, reduced Nrf2 levels and down-regulation of Nrf2 target gene products, including HO-1 and NQO-1, in the hippocampus (Si et al., 2023). Notably, Nrf2 activity declines with aging (Suh et al., 2004), thus implying that elderly individuals may be more vulnerable to the detrimental effects of social isolation as a consequence of decreased antioxidant defenses.

Oxidative stress and inflammation are closely interconnected processes. NF-κB has been shown to act as a negative regulator of Nrf2 (Liu et al., 2008), and vice versa Nrf2 knock-out animal or cellular models displayed enhanced NF-κB activation and upregulation of downstream pro-inflammatory target genes such as TNF-α, IL-1β, and IL-6 after injury (Jin et al., 2008; Pan et al., 2012), suggesting that defective Nrf2 signaling may exacerbate inflammatory responses in brain cells exposed to stressors. Accordingly, adult male rats subjected to social isolation for 5 weeks showed a decreased expression of Nrf2 and antioxidant proteins with a reciprocal increased expression of NF-κB and inflammatory mediators (TNF-α, IL-1β, IL-6, prostaglandin E2) in brain tissue. Treatment with the natural antioxidant polyphenol punicalagin during the isolation period reverted these molecular phenotypes and restored the behavioral alterations associated with social isolation (Salem et al., 2023). Along the same line, male rats subjected to chronic social isolation displayed high levels of NF-κB in the hippocampus as compared to controls. The administration of resveratrol, a potent Nrf2 inducer (Zamanian et al., 2023), attenuated NF-κB activation and behavioral alterations in isolated animals (Zarebavani et al., 2023).

Interestingly, oxidative stress and hypofunctional Nrf2 have been suggested to contribute to metabolic diseases and associated cardiovascular risk (da Costa et al., 2019). As such, Nrf2-directed antioxidant treatments represent a promising therapeutic strategy to counterbalance both the neurological and metabolic outcomes of social isolation.

### Social isolation induces organelle stress

#### Endoplasmic reticulum stress

The endoplasmic reticulum (ER) has a specialized oxidized environment that allows proper protein folding, making the ER extremely sensitive to changes in cellular redox states (Cao and Kaufman, 2014). Oxidative stress and ER stress interact in a complex chain of events in many metabolic and inflammatory disorders. Prolonged oxidative stress can evoke ER stress by changing the cellular redox state, which disrupts calcium signaling and protein folding, initiating pathways known as the unfolded protein response (UPR). The UPR involves three main membrane-associated proteins: PERK (PKR-like endoplasmic reticulum kinase of the eukaryotic initiation factor 2α, eIF2α), IRE1 (inositol requiring enzyme 1), and ATF6 (activating transcription factor-6). Under basal conditions, these three proteins are kept inactive through their interaction with the chaperone BiP (or GRP78), which dissociates upon UPR activation. Overall, the activation of the three UPR branches leads to inhibition of general protein synthesis, upregulation of genes involved in protein folding and degradation, as well as activation of inflammatory signaling and further ROS production. If the stress persists, the UPR can ultimately trigger apoptosis through effectors such as CHOP (Hotamisligil, 2010).

Although very little is known, the available evidence suggests that ER stress responses might be triggered in the brain of social isolation models. In *Drosophila melanogaster*, isolation for 7 days reduced sleep, impaired memory and caused an induction of the UPR. At the molecular level, the brains of isolated flies showed an increase of BiP levels, XBP1 mRNA splicing and phosphorylation of eIF2α. Interestingly, regrouping the flies restored normal BiP levels (Brown et al., 2017). In another study, adult rats socially isolated for 5 weeks showed a marked induction of PERK, GRP78 and CHOP mRNAs in whole brain homogenates, suggesting the activation of ER stress responses (Salem et al., 2023).

#### Mitochondrial dysfunction

Several reports suggest that mitochondria are affected in socially isolated mice and rats, with an impact on ATP synthesis, ROS production, and apoptosis regulation, even though contrasting results have been obtained depending on the species, brain region and isolation paradigm. Young rats isolated for 30 days displayed a reduced mitochondrial volume in neurons of the visual cortex (Sirevaag and Greenough, 1987). Mitochondrial respiratory complexes, responsible for ATP production, were found altered in different brain regions. Reduced activity of the respiratory complexes I, II, and IV has been described in the PFC (Filipović et al., 2011, 2023; Al Omran et al., 2022), although some authors reported no effects or even increased activity (Adzic et al., 2009; Krolow et al., 2012). In the hippocampus, increased COX-IV levels in synaptic mitochondria have been reported in isolated mice (Zhang et al., 2012), whereas no change was detected in adult rats (Filipović et al., 2011). In addition, social isolation was associated with decreased levels of proteins involved the TCA cycle (Aco2) and oxidative phosphorylation (Uqcrc2, Atp5f1a, and Atp5f1b) in non-synaptic mitochondria of the rat PFC (Filipović et al., 2020, 2023). Lower ATP levels were consistently detected in isolated mice and rats in the hippocampus, PFC and nucleus accumbens (Möller et al., 2013; Silva et al., 2020; Watanabe et al., 2022). Interestingly, some of these changes appear to be reversible upon pharmacological treatment with the anti-psychotic clozapine (Möller et al., 2013), the anti-depressant fluoxetine (Filipović et al., 2020), or with antioxidants such as resveratrol (Zhao et al., 2022) or N-acetylcysteine (Möller et al., 2013).

Mitochondria are one of the main sources of ROS, and in turn mitochondrial proteins are highly sensitive to oxidative damage. Young rats isolated for 30 days showed reduced activity of aconitase, creatine kinase, and succinate dehydrogenase in hippocampal mitochondria, indicating mitochondrial oxidative damage, and increased hexokinase activity, suggesting a possible shift toward anaerobic glycolysis (Zhuravliova et al., 2009). Decreased levels of the mitochondrial ROS scavenging enzyme superoxide dismutase (SOD) were found in the PFC, but not in the hippocampus of isolated rats (Filipović et al., 2011). Others, however, found increased SOD activity in the PFC of rats after 1 week of post-weaning isolation, and this effect lasted after the end of the isolation in their adult life (Krolow et al., 2012). Two-week social isolation in adult mice increased ROS and several mitochondrial antioxidant enzymes, including SOD2, HO-1, PRDX-3, and GPX4 in the PFC (Al Omran et al., 2022).

Social isolation probably has an effect also on apoptosis regulation by mitochondria, although results are conflicting. Social isolation in young/adult rats induced variable perturbations in the levels of mitochondrial *vs* cytoplasmic Bax (pro-apoptotic member of the Bcl2 family of proteins) and Bcl2 (anti-apoptotic protein) in the hippocampus and PFC (Djordjevic et al., 2009; Adzic et al., 2009; Djordjevic A. et al., 2010; Djordjevic et al., 2012a; Djordjevic et al., 2012b; Zlatković and Filipović, 2012). Moreover, cleaved caspase-3 was detected in the PFC of adult isolated rats, suggesting the activation of pro-apoptotic signaling (Filipović et al., 2011).

#### Autophagy dysfunction

Macroautophagy (hereafter autophagy) is a highly conserved catabolic process through which cells degrade damaged intracellular components such as aggregated proteins and dysfunctional organelles into the lysosome to recycle cellular components, according to the metabolic status of the cell and in response to various stressors (González et al., 2020).

Male mice isolated for 15–25 weeks displayed increased hippocampal levels of phosphorylated AKT and mTOR, which inhibit the autophagic pathway (Martina et al., 2012). Accordingly, the levels of the autophagic factors Beclin1 and LC3B-II were reduced, while the levels of the autophagic receptor p62/SQSTM1 were increased, consistent with an inhibition of the autophagic pathway in long-term isolated animals (Wang et al., 2019, 2022). These changes were accompanied by alterations in the levels of postsynaptic proteins, as well as by impaired long-term potentiation of hippocampal synaptic transmission. Notably, these molecular and functional synaptic deficits were fully rescued by a two-week treatment with rapamycin, an mTOR inhibitor and autophagy inducer (Wang et al., 2019).

In addition, oxidative stress plays a crucial role in the regulation of autophagy as a means of maintaining redox balance. ROS have been reported to activate autophagy through the S-glutathionylation of AMPK (Filomeni et al., 2015). However, pathological ROS production may lead to autophagy inhibition through the oxidation of other factors, such as ATG3 and ATG7, implicated in autophagosome maturation (Frudd et al., 2018). Nrf2 promotes the transcription of various autophagy genes (e.g., SQSTM1, ATG2B, ATG4D, ATG5, ATG7) as part of the antioxidant response (Pajares et al., 2016). Moreover, a synergistic interplay between Nrf2 and autophagy through a p62/SQSTM1–Keap1–Nrf2 axis has been described (Ichimura et al., 2013). As previously mentioned, defective Nrf2 signaling has been described in social isolation, and this may in turn contribute to autophagy dysfunction in isolated animals.

## Conclusion

**Figure 1:**
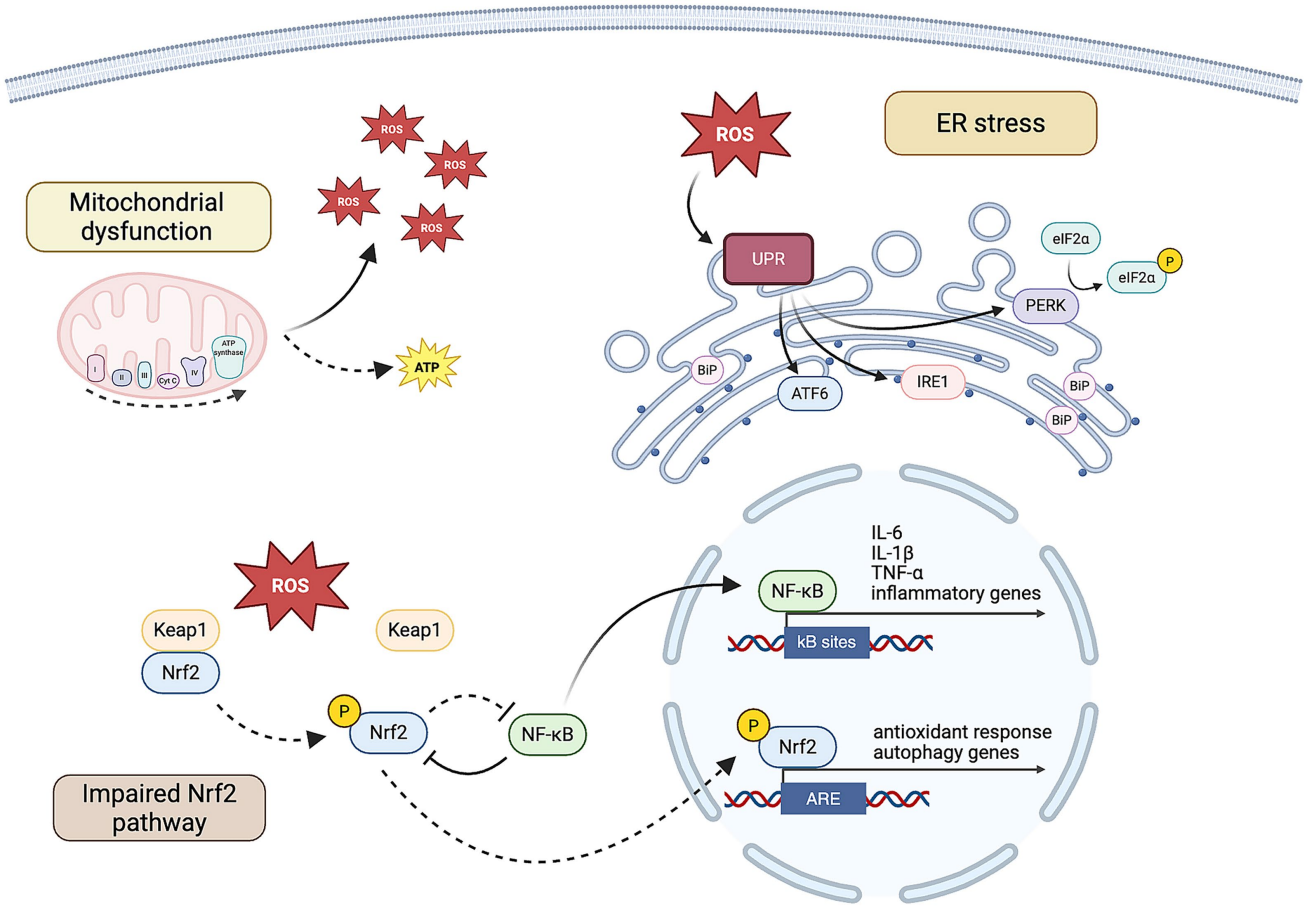
Molecular pathways deregulated in the context of social isolation. Mitochondrial dysfunctions and alterations in the respiratory chain lead to decreased ATP production, increased ROS generation and oxidative stress. ROS may contribute to the induction of the three branches of the UPR in the ER. The chaperone BiP dissociates from its partner proteins: PERK phosphorylates eIF2α, with subsequent inhibition of protein synthesis; IRE-1 and ATF6 activate a transcriptional program that promotes inflammatory and pro-apoptotic signaling. The antioxidant Nrf2 pathway is impaired: despite ROS promote its detachment from Keap1, the Nrf2 pathway is suppressed and fail to induce proper antioxidant response and autophagy. NF-κB signaling prevails, promoting the expression of inflammatory mediators. Created in BioRender (https://BioRender.com/8fy05d1).

Social isolation and loneliness have emerged as a public health concern in modern societies, further exacerbated by the COVID-19 pandemic and its long-lasting social repercussions (Su et al., 2023). A growing body of clinical evidence links social isolation and loneliness not only to psychological distress but also to a heightened risk of MetS, CVD and premature mortality. Insights from experimental models have been instrumental in identifying oxidative stress, chronic inflammation, ER stress, mitochondrial dysfunction, and impaired autophagy as pathophysiological processes implicated in social isolation (Figure 1). These interconnected cellular stress pathways disrupt metabolic homeostasis and promote a cascade of events that probably contribute to brain dysfunctions and systemic complications. Although the precise interactions among these pathways remain to be fully clarified, they represent promising targets for therapeutic intervention. Advancing our understanding of these molecular mechanisms is crucial for mitigating the health consequences of social disconnection and for developing comprehensive strategies to address its rising impact on global public health.

## Group members of the SOLITAIRE group

Marcella Bellani, Maria Gloria Rossetti, Cinzia Perlini, Francesca Girelli, Niccolò Zovetti, Maria Diletta Buio: Department of Neurosciences, Biomedicine and Movement Sciences, University of Verona, Verona, Italy & Azienda Ospedaliera Universitaria Integrata Verona, Verona, Italy; Paolo Brambilla, Cinzia Bressi, Antonella delle Fave, Virginia Pupi: Department of Pathophysiology and Transplantation, University of Milan, Milan, Italy; Fabrizia Claudia Guarnieri, Edoardo Moretto: CNR Institute of Neuroscience, Vedano al Lambro, Italy; Roberto Sassi, Maria Renata Guarneri, Stavros Ntalampiras, Samara Soares Leal: Department of Computer Science, University of Milan, Milan, Italy; Isabella Fanizza, Lara Scialpi, Giorgia Carlucci, Mariangela Leucci: Unit for Severe Disabilities in Developmental Age and Young Adults, Associazione La Nostra Famiglia - IRCCS *E. Medea*, Scientific Hospital for Neurorehabilitation, Brindisi, Italy; Antonio Trabacca: Scientific Institute IRCCS Eugenio Medea, Scientific Direction, Bosisio Parini, Lecco, Italy.
